# Malignant neuroendocrine tumour of the appendix in childhood with loco-regional lymph node invasion

**DOI:** 10.1186/s13000-015-0287-z

**Published:** 2015-05-29

**Authors:** Rebecca F Lyons, Muhammad Irfan, Ronan Waldron, Niamh Bambury, Fadel Bennani, Tamas Nemeth, Waqar Khan, Kevin Barry

**Affiliations:** Department of Surgery, Mayo General Hospital, Saolta Hospital Group, Mayo, Ireland; Department of Histopathology, Mayo General Hospital, Saolta Hospital Group, Mayo, Ireland; Professor of Surgery, Discipline of Surgery, National University of Ireland, Galway, Ireland

**Keywords:** Neuroendocrine tumours, Childhood, Metastatic, Lymph node

## Abstract

The virtual slide(s) for this article can be found here: http://www.diagnosticpathology.diagnomx.eu/vs/1006600359152743

**Electronic supplementary material:**

The online version of this article (doi:10.1186/s13000-015-0287-z) contains supplementary material, which is available to authorized users.

## Background

Malignant neuroendocrine tumour of the appendix is a rare finding in the paediatric population. Metastases to the loco-regional lymph nodes at time of diagnosis are very rare. The literature reviewing the treatment and follow up of such cases is not definitive regarding best practice and management guidelines vary due to the complexity and rarity of these tumours.

## Case presentation

A twelve year old girl presented acutely to the emergency department, with a two day history of right iliac fossa pain associated with nausea and vomiting. No fevers or rigors were reported. The patient denied urinary symptoms and was pre-menstrual. She had a background of recurrent presentations to the emergency department with non specific abdominal pain in the preceding months. This girl had also been investigated by the paediatric service regarding episodes of dizziness, headaches and recurrent epistaxis. All previous investigations were normal. On this occasion, she had tenderness, localised guarding and rebound in the right iliac fossa. Her inflammatory markers were elevated, white cell count (WCC) 14.2 and a C reactive protein (CRP) of 23.4. Intravenous antibiotics were commenced and a plan for surgical intervention was made.

**Figure 1:**
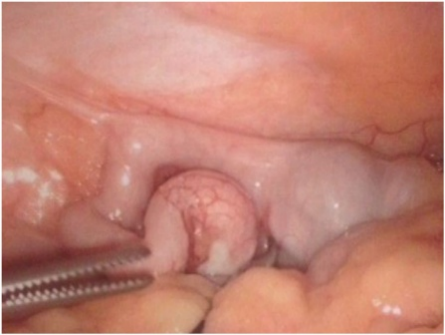
Intra-operative image of the appendix at initial operation.

The patient underwent an emergency uncomplicated laparoscopic appendicectomy. At the time of surgery, it was noted that the apex of the appendix was distended. (Figure 1) There were no signs of acute inflammation or purulent fluid. The base of the appendix was ligated using endoloops and the specimen was submitted for histology. The patient made an uncomplicated recovery and was discharged forty eight hours later.

**Figure 2:**
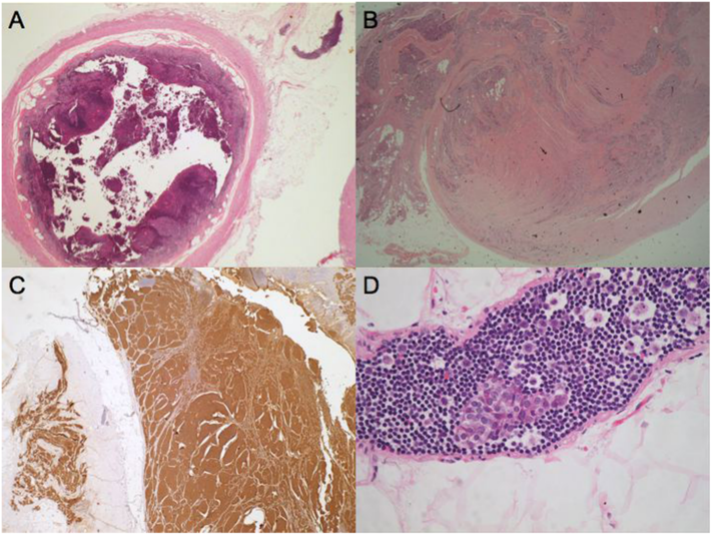
Highlights the histological appendix specimen with staining. **A**: Base of the appendix with accompanying mesoappendiceal lymph node. **B**: Neuroendocrine carcinoma of the appendix located at the apex with extramural extension into the mesoappendix. **C**: Chromogranin A, neuroendocrine marker positive. **D**: Mesoappendiceal lymph node showing small focus of metastatic tumour.

**Figure 3:**
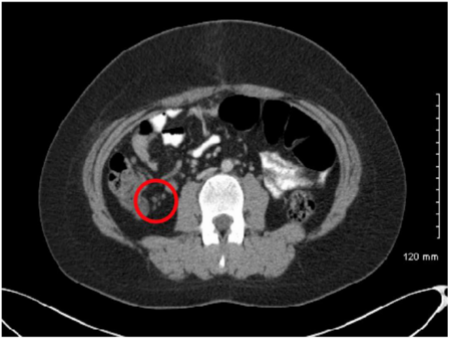
CT scan identifying the sub centimetre lymph nodes in right paracolic gutter.

Pathological examination of the appendix (measuring 60 mm in length) demonstrated a rare case of infiltrating neuroendocrine tumour of the appendix. The tumour penetrated the mucosa, submucosa and muscularis propria with extraluminal extenstion into the mesoappendix. This tumour was well differentiated measuring 25 mm in maximal diameter. The tumour stained positive for chromogranin, CD 56 and synaptophysin. The Ki67 proliferative index measured 15%, therefore consistent with a Grade 2 (G2) neuroendocrine tumour. A microscopic lymph node of the mesoappendix was also analysed which stained positive for chromogranin A, confirming a small metastatic deposit in the centre of the lymph node*.* (Figure 2) The provisional staging of pT3, N1 Mx was assigned and the patient proceeded to staging investigations. A computerised tomography (CT) scan of the thorax, abdomen and pelvis was performed demonstrating a cluster of sub-centimetre lymph nodes in the right paracolic gutter of indeterminate significance. (Figure 3) Urinary 5 HIAA testing was also completed and a normal level reported.

**Figure 4:**
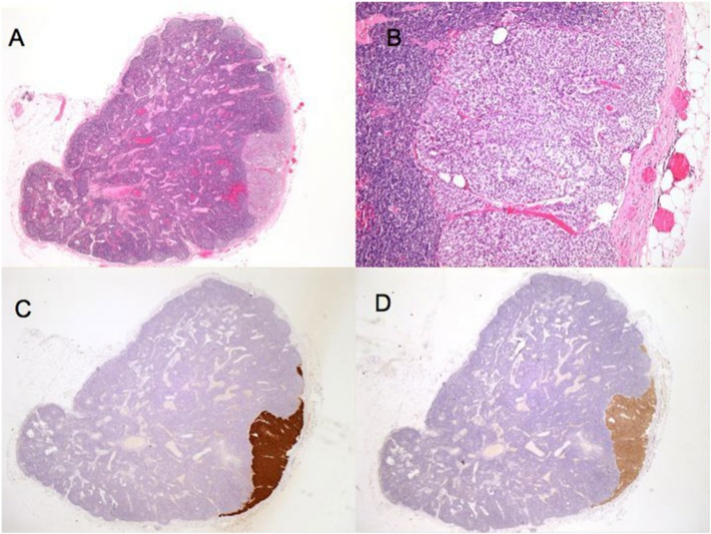
Shows the histological lymph node specimen with staining. **A** and **B**: Large lymph node from right hemicolectomy specimen showing metastatic tumour. **C** and **D**: Immuno staining positive for chromogranin A (C) and synaptophysin (D).

After discussion at the gastrointestinal multidisciplinary meeting, the decision to perform a laparoscopic right hemicolectomy was made. The pathological examination of the subsequent right hemicolectomy specimen showed no evidence of residual tumour grossly or microscopically. Forty seven loco-regional lymph nodes were retrieved. Two of these lymph nodes were positive for metastatic neuroendocrine tumour. These were located at 1 cm and 5 cm from the appendicectomy site. All peri-ileal lymph nodes were free of disease. The immunohistochemistry profile again was positive for chromogranin, synaptophysin and CD 56. (Figure 4).

The patient is currently doing well and has been enrolled on a clinical and magnetic resonance imaging (MRI) surveillance programme.

## Conclusions

Laparoscopic appendicectomy is a common procedure in the paediatric age group. Rarely has an unexpected diagnosis of a malignant neuroendocrine tumour of the appendix been noted. Interestingly benign neuroendocrine tumours are the most common tumour of the gastrointestinal tract in children, however malignant neuroendocrine tumours are rare [1]. Table 1 highlights the key publications on malignant neuroendocrine tumours within the paediatric population. Definitive incidence reporting across the literature is lacking, with varying incidences being reported. However, Parkes et al. [2] has been referenced widely in the literature with an incidence of 1.14 per million children. The other large study by Boxberger et al. concluded that the incidence was 1 per 100,000 children, a value that is widely referenced in the literature.

**Table 1:** Highlights the incidence of malignant neuroendocrine tumours in the literature

**Author**	**Number of children in series**	**Incidence reported**
Parkes et al. 1993 [2]	40 children over 24 years	1.14 per million children
Pelizzo et al. 2001 [1]	10 children over 8 years	1.14 per million children
D’Aleo et al. 2001 [15]	2 children	1 per 100,000 children
O’Donnell et al. 2006 [13]	3 children over 9 years	1 per 100,000 children
Boxberger et al. 2013 [3]	237 children over 5 years	1 per 100,000 children

Up to 90% are diagnosed incidentally after laparoscopic appendicectomy [1]. Usually there is a large female predominance [3]. This is interesting, when one considers the recent trend in the literature looking at the conservative management of acute appendicitis in children [4].

The presenting features of both benign and malignant neuroendocrine tumours usually follow that of acute appendicitis as highlighted in our case. The well described carcinoid syndrome of flushing, diarrhoea and cardiac disease is rarely reported within the paediatric population as this is associated with liver or retroperitoneal metastases. It is in such cases that the 5 hydroxyindoleacetic acid (5HIAA) testing is positive [1,3,5,6].

Boxberger *et. al* studied neuroendocrine tumours in children over a five year period. They noted that mean age of presentation was 13 yrs (4.5-19.5), the majority of those presented with signs of acute appendicitis and the diagnosis was made histologically. The location of the tumour similar to our case primarily was at the apex of the appendix (70%) with extension into the mesoappendix in 63%. Extension into the mesoappendix was more likely if the size of the primary tumour was over 15 mm.

It has been confirmed across the literature that site, size and grade are significant in predicting aggressive behaviour of tumours [1,7]. Prognosis has been found to be directly related to tumour size. Rossi et al. questioned whether or not mesoappendiceal involvement was an indicator or poor prognosis, however their study confirmed previous studies findings, that size is the main determinant of prognosis [8,9,10].

Decision on further operative treatment after histological confirmation of malignant neuroendocrine tumours is based on the size of the tumour. If the tumour is less than 2 cm appendicectomy alone is the operation of choice. A low proliferative index, an apical location of the tumour, and lack of angiolymphatic or mesoappendiceal invasion are other factors that influence surgery. A right hemicolectomy is the operation of choice if the tumour is greater than 2 cm, or if there is histological evidence of mesoappendiceal extension or location of the tumour at the base with caecal extension. However it must be noted that only 20% of resected specimens will show any residual disease.

The World Health Organisation revised the classification system for neuroendocrine tumours in 2010 and places considerable emphasis on the Ki67 proliferative index. The Ki67 index is used to subdivide the neuroendocrine tumours into G1 or G2 neoplasms. If the Ki67 index is less than 3%, these are classified as G1. A Ki67 index between 3-20% classifies the tumours as G2. G3 is represented by a Ki67 greater than 20%. Ki67% has been studied as a factor for predicting metastases or recurrence. In 2013 Yamaguchi *et. al* investigated Ki67 as a predictive index of tumour spread. This important study reported that a Ki67 index of 2.8% or greater gave a specificity of 86.8% of having metastases or recurrence [11]. When assessing Ki67 as a marker for the biologic behaviour of tumours, it must be considered that Ki67 expression varies during the disease progression. This is not fully understood at present, but literature available suggests that Ki67 expression does vary and depending on the time of measurement, Ki67 can result in the WHO classification being upgraded. This has significant implications for treatment and follow up of these patients [12].

The presence of lymph node involvement as in our case, is rare and has been reported sporadically in 4-5% of paediatric cases [13,6]. A review of 414 cases looking at neuroendocrine tumour and metastases found that only 4.1% of the cases had metastases identified. MacGillivary *et. al* confirmed that tumours greater than 2 cm and mesoappendiceal invasion are associated with metastatic disease [6,14]. D’Aleo *et. al* suggested that a right hemicolectomy for a child with a neuroendocrine tumour of the appendix is a radical procedure as the prognosis is quite good. 5 year survival is reported between 90-100%. There is a trend towards limited resection as an alternative option to the classic right hemicolectomy, with perhaps an ileocaecal resection deemed appropriate [1].

No definitive follow up has been quoted in the literature. Despite the incidence of recurrent disease being low, follow up is recommended. In general terms clinical follow up, including chromogranin A (CgA) and 5 HIAA testing is recommended. No studies have assessed the sensitivity of these biologic markers to detect metastases or local recurrence [3,15,16]. The ENETS guidelines recommend that if the tumour is less than 1 cm then no specific follow up is needed. However if there is involvement of lymph nodes, long term follow up is recommended. MRI or CT is recommended in cases where the initial tumour is greater than 2 cm, local invasion or metastatic disease are present at diagnosis. MRI should be considered in the young and in females of childbearing age due to the lower radiation doses when compared to serial CT scanning. It is recommended that these high risk patients are followed up at 6 months and 12 months post operatively and annually thereafter.

## Consent

Consent was obtained from the patient for publication.
